# Understanding and Addressing Mental Health Stigma Across Cultures for Improving Psychiatric Care: A Narrative Review

**DOI:** 10.7759/cureus.39549

**Published:** 2023-05-26

**Authors:** Ahmed A Ahad, Marcos Sanchez-Gonzalez, Patricia Junquera

**Affiliations:** 1 Psychiatry and Behavioral Sciences, Florida International University, Herbert Wertheim College of Medicine, Miami, USA; 2 Health Services Administration, Lake Erie College of Osteopathic Medicine, Bradenton, USA

**Keywords:** psychology, educational interventions, mental health services, cultural differences, ethnicity, psychiatry, mental health, stigma

## Abstract

Stigma, characterized by negative stereotypes, prejudice, and discrimination, is a significant impediment in psychiatric care, deterring the timely provision of this care and hindering optimal health outcomes. Pervasive in all aspects of psychiatric care, stigma leads to delayed treatment, increased morbidity, and diminished quality of life for those with poor mental health. Hence, better understanding the impact of stigma across different cultural contexts is critically essential, aiming to inform culturally nuanced strategies to minimize its consequences and contribute to a more equitable and effective psychiatric care system. The purpose of the present literature review is twofold (i) to examine the existing research on the stigma surrounding psychiatry across different cultural contexts and (ii) to identify the commonalities and differences in the nature, magnitude, and consequences of this stigma in different cultures in the psychiatry field. In addition, potential strategies for addressing stigma will be proposed. The review covers a range of countries and cultural settings, emphasizing the importance of understanding cultural nuances to combat stigma and promote mental health awareness globally.

## Introduction and background

Stigma, characterized by societal prejudice and discrimination, profoundly influences psychiatric care, creating barriers to the timely recognition and treatment of mental health disorders [1]. Deeply embedded in societal norms, stigma is a multifaceted issue permeating every level of psychiatric care, leading to delayed treatment, increased morbidity, and a diminished quality of life for patients.

The importance of addressing stigma in psychiatry cannot be overstated as stigma impacts individuals seeking care, their families, healthcare professionals, and broader society. At the individual level, stigma can lead to fear and avoidance of mental health services, causing delays in seeking help even when a patient is in dire need. Delays in seeking care can exacerbate mental health conditions leading to worse outcomes and reduced quality of life [2]. For families, the stigma can lead to shame and isolation, making seeking necessary support and resources more difficult. Interestingly, in healthcare professionals, stigma can lead to burnout and demoralization, reducing the quality and provision of care. Stigmatization can also create barriers between healthcare providers and patients, complicating matters to establishing trustful and therapeutic relationships, which are essential for effective care [1]. For society at large, stigma can result in the misallocation of resources, with mental health services often being underfunded and overlooked [3]. Hence stigma has profound effects at personal and societal levels, negatively impacting multiple levels of the psychotic care continuum. 

Addressing the stigma surrounding mental health can significantly enhance the effectiveness of psychiatric care. To this end, developing programs and strategies that foster a culture of understanding and acceptance may encourage more individuals to seek help when they need it, improving early detection and intervention, which are crucial for better health outcomes. Furthermore, challenging and changing stigmatizing attitudes can improve the therapeutic relationship between healthcare providers and patients, leading to more personalized and effective treatment strategies.

Stigma, however, is not a monolithic entity but varies across cultures, influenced by distinct societal norms, values, and beliefs. Understanding these cultural variations is essential for developing effective, culturally sensitive interventions. Therefore, this literature review aims to examine the manifestation and impacts of stigma across different cultural contexts, laying the foundation for tailored strategies to combat this healthcare barrier.

## Review

Stigma as a psychological construct

In the literature, there have been several attempts at creating instruments to measure and understand stigma as a psychological construct in the context of mental health. In this vein, the Internalized Stigma of Mental Illness (ISMI) scale and the Perceived Devaluation-Discrimination Scale, among others, seek to quantify stigma more objectively [4,5]**.** The ISMI scale, as defined by Ritsher et al. (2003), measures the subjective experience of stigma, including the internalization of negative stereotypes and beliefs about mental illness [4]. It includes five subscales: Alienation, Stereotype Endorsement, Discrimination Experience, Social Withdrawal, and Stigma Resistance. These subscales were further defined as follows: (i) Alienation: The feeling of being less than a full member of society due to one's mental illness, (ii) Stereotype Endorsement: The extent to which the individual agrees with common negative stereotypes about people with mental illness, (iii) Discrimination Experience: Personal experiences of rejection or exclusion due to mental illness, (iv) Social Withdrawal: The extent to which the individual avoids social situations for fear of being stigmatized, and (v) Stigma Resistance: The individual's ability to resist or counteract stigma. The Perceived Devaluation-Discrimination Scale, as described by Link (1987), measures the extent to which individuals believe that most people will devalue or discriminate against someone with a mental illness [5]. It focuses on the individual's perceptions of societal attitudes, rather than their personal experiences with stigma. Overall, while the ISMI scale can give insights into the internalization and personal experience of stigma, the Perceived Devaluation-Discrimination Scale can provide a view of societal attitudes and perceived discrimination. The above are crucial to understanding the full landscape of stigma in psychiatry across different cultures by helping identify where interventions might be most needed and most effective, whether at the level of societal attitudes, personal beliefs, or both. The pervasive nature of stigma presents a daunting challenge to psychiatry, necessitating a rigorous and nuanced approach to its understanding and mitigation. However, despite recent awareness campaigns, the field still struggles with the barriers that stigma imposes on patient care, necessitating additional analysis of the effects.

Individual and societal impact of stigma

Stigmatization of mental illness across cultures is a significant barrier to psychiatric care. The stigma can lead to delayed diagnosis and treatment-seeking behaviors, reduced quality of life, and an increased risk of social exclusion and discrimination [2]. Furthermore, mental illness stigma often intersects with other forms of stigma, such as gender, race, and socio-economic status, leading to further marginalization of already vulnerable populations making it challenging to provide equitable, culturally sensitive, and effective psychiatric care to individuals with mental illness. Accumulating research suggests that stigma toward mental illness is common in various cultures, which can affect mental illness diagnosis, treatment, and management [6]. Furthermore, some studies reveal that mental health stigma manifests differently across cultures and can be influenced by cultural beliefs, attitudes, and values [7]. The stigma surrounding psychiatry and mental health disorders has numerous detrimental effects on individuals and communities, including:

1. Delayed Treatment-Seeking Behavior

Stigma plays a significant role in delaying treatment-seeking behavior for individuals struggling with mental health issues. The fear of being labeled, ostracized, or misunderstood due to their condition often deters individuals from seeking help promptly. According to a study by Clement et al. (2015), stigma was associated with an increased likelihood of delaying or avoiding seeking help for mental health concerns [8]. Consequently, symptoms may worsen over time, escalating the condition's severity and making treatment and prospective recovery more challenging. Healthcare delays can also lead to decreased self-esteem and increased depressive symptoms, creating a vicious cycle of self-blame, isolation, and hopelessness. Prolonged untreated mental health issues can further impair an individual's functionality in various life domains, including work, relationships, and self-care, thus reducing their overall quality of life [9].

2. Social Isolation and Discrimination

Stigma can lead to social isolation and discrimination for those affected by mental health issues. Brohan and Thornicroft (2010) found that individuals with mental health disorders often face discrimination in multiple life domains, including employment and interpersonal relationships [2]. The negative stereotypes and misconceptions surrounding mental illness often result in a lack of understanding and empathy from others, leading to social exclusion [10]. Individuals with mental health issues might face discrimination in various aspects of life, including the workplace, where they might encounter bias in hiring, job retention, and career advancement. Furthermore, to complicate matters, discrimination can further strain personal relationships, as friends and family may distance themselves due to discomfort, fear, or misunderstanding, exacerbating feelings of isolation and loneliness [9].

3. Reduced Treatment Adherence

Stigma can significantly impact adherence to mental health treatments. Sirey et al. (2001) found that perceived stigma predicted treatment discontinuation in older adults with depression [11]. People living with mental health conditions may avoid or discontinue treatment due to fear of being identified as a mental health patient. This fear could stem from concerns about the stigma associated with visiting mental health facilities, taking psychiatric medications, or being seen engaging in therapeutic activities [12]. Non-adherence to treatment regimens can lead to suboptimal treatment outcomes, hinder recovery, and increase the risk of relapse or worsening symptoms. Furthermore, stigma can diminish self-efficacy, making individuals less likely to actively engage in their treatment process, which is crucial for successful recovery.

4. Perpetuation of Misconceptions

Stigmatizing attitudes towards mental illness contribute to the perpetuation of harmful stereotypes and misinformation. AsCorrigan and Watson (2007) discussed, stereotypes such as appearing dangerous, unpredictable, or culpable for their illness can make people with mental illness perceived inaccurately as dangerous or to blame for their condition, both internally and externally [12]. Stereotyping, deeply embedded in societal attitudes, can foster a culture of fear, rejection, and discrimination against individuals with mental health conditions. Misconceptions often result in people with mental health issues being perceived inaccurately as dangerous, unpredictable, or responsible for their condition. In addition, misinformation can hinder public understanding and acceptance of mental illness, exacerbating stigma while negatively influencing policy and legislation, leading to inadequate funding and support for mental health services.

5. Influence of Gender on Stigma

The impact of stigma on individuals with mental illness is known to vary across different social and demographic categories, including gender. Research evidence indicates that the experience of stigma related to mental illness can be significantly different for men and women, and these differences can be further influenced by cultural context.

In some societies, women seem to face higher levels of stigma related to mental health issues compared with men. A study by Al Krenawi et al. (2006) conducted in the Bedouin-Arab community found that women experienced a significantly higher degree of stigma associated with mental illness than their male counterparts [13]. This may be due to traditional gender roles and societal expectations, which often place women in a more subordinate position and associate mental illness with weakness or vulnerability. Women with mental illnesses may therefore face dual discrimination - first for their gender and then for their mental health condition. This can make women less likely to seek help for mental health issues, further exacerbating their condition and creating a vicious cycle of stigma and untreated mental illness.

However, the influence of gender on stigma is not uniform across all cultures. Ayalon and Areán's (2004) study on older adults in an Arab cultural context found that men reported higher levels of perceived stigma related to mental illness than women [14]. This discrepancy might be rooted in traditional masculine norms prevalent in many Arab societies, which value strength, stoicism, and emotional control. Mental illness, which is often erroneously perceived as a sign of emotional weakness or lack of control, can be particularly stigmatizing for men in these contexts. Furthermore, the expectation for men to be the primary earners and providers in the family can make the potential economic impacts of mental illness, such as unemployment or reduced productivity, particularly stigmatizing.

These findings underscore the importance of considering gender and cultural context in understanding and addressing stigma related to mental illness. It is crucial to develop and implement culturally sensitive strategies that consider these differences in the experience of stigma. This might involve, for example, promoting mental health literacy, challenging harmful gender norms, and providing gender-specific mental health services. We can move toward a more equitable and effective mental health care system by acknowledging and addressing the unique stigma-related challenges different groups face.

Ethnic and cultural variations in stigma

The stigma surrounding psychiatry, as research suggests, manifests differently across cultures due to various factors [7]. This stigma operates at various levels, including individuals, families, healthcare providers, and society, and cultural norms, religious beliefs, and social attitudes influence its manifestations and implications.

At the individual level, mental health issues may be internalized differently depending on cultural background. For instance, some Asian cultures may view mental health issues as a sign of personal weakness or a failure of self-control [15]. The internalization of stigma can significantly influence an individual's self-perception and willingness to seek help. In the family context, cultural beliefs also play a significant role in shaping attitudes toward mental health. A study by Yang and Kleinman (2008) found that in Chinese culture, mental illness is often attributed to social and interpersonal factors, such as family conflict [16]. Such attributions can contribute to a sense of shame or blame within the family, exacerbating the stigma experienced by the individual with mental illness.

Healthcare providers are not immune to these cultural beliefs and they can influence their practice. In some cultures, mental illnesses are viewed through a supernatural lens rather than a medical one. Girma et al. (2013) found that in Ethiopian culture, mental illness is commonly associated with supernatural causes, such as evil spirits or curses [17]. This widely held belief can influence healthcare providers' approach and potentially limit the provision of evidence-based psychiatric care.

Lastly, at the societal level, these cultural perceptions and beliefs can contribute to the broader social stigma surrounding mental health, leading to discrimination and social exclusion. Differences in societal perceptions across cultures can lead to distinct forms of discrimination, further compounding the challenges faced by individuals with mental health issues. Hence, understanding and addressing cultural stigma in psychiatry involves a multifaceted approach that considers individual, family, healthcare providers, and societal levels. Each level offers potential avenues for stigma reduction and improved mental health outcomes.

Asian Cultures

In many Asian societies, mental health issues are often perceived as a sign of personal weakness or a failure of self-control. The concept of 'face' is significantly influential, and the stigma associated with mental illness can be seen as bringing shame to the family [15]. For instance, a strong cultural emphasis on academic and professional achievement in South Korea contributes to stigmatizing attitudes toward mental illness, which may discourage individuals from seeking help [18].

African Cultures

Mental illnesses in some African cultures are often attributed to spiritual or supernatural causes such as curses or possession by evil spirits. This understanding can contribute to high levels of stigma and deter individuals from seeking psychiatric help [19]. In Ethiopia, the belief in supernatural causes of mental illness has been reported, leading to the stigmatization of affected individuals [17].

Arab Cultures

Mental illness in Arab societies is frequently viewed as a form of divine punishment. Religious belief perpetuating mental health stigma can lead to delayed or avoided treatment as individuals may resort to religious or spiritual interventions [20].

Latin American Cultures

In some Latin American cultures, mental illness is often attributed to personal weakness or lack of willpower. This perspective could stigmatize individuals with mental health disorders and discourage them from seeking psychiatric care [21].

Western Cultures

In Western societies, stigma often stems from misconceptions about mental illness, including the belief that individuals with mental health disorders are dangerous or unpredictable. While mental illness is recognized more as a health issue, stigma still exists, often resulting in social exclusion and discrimination [12].

Additionally, culture-bound syndromes, defined here as a combination of psychiatric and somatic symptoms that are considered to be a recognizable disease within specific cultures or societies, are a critical component of a discussion on cultural stigma in psychiatry. That is to say, culture-bound syndromes refer to unique mental health conditions closely tied to specific cultures or ethnic groups. For instance, among the Latino community, 'Ataque de Nervios,' characterized by uncontrollable shouting, crying, trembling, and sometimes aggressive behavior, is a recognized condition often associated with a stressful event such as a panic attack [21].

Hence, a clinician's awareness and understanding of such culture-bound syndromes can enhance their diagnostic and therapeutic effectiveness. In fact, a study conducted by Hughes and Wintrob (1995) in New York discovered a significant improvement in therapeutic relationships when clinicians were knowledgeable about culture-bound syndromes prevalent in their patients' cultures, such as 'Qigong Psychotic Reaction' in Chinese immigrants, a condition associated with overdoing Qigong, a type of spiritual martial art [22].

Furthermore, cultural competence, which includes knowledge about culture-bound syndromes, has a substantial impact on treatment outcomes. Culturally competent care, defined by an understanding and respect for cultural differences, can improve patient satisfaction and adherence to treatment. A systematic review by Truong et al. (2014) demonstrated the positive effect of cultural competence on healthcare outcomes, including in a Native American population suffering from 'Ghost Sickness,' a culture-bound syndrome characterized by feelings of terror, weakness, and a sense of impending doom, often linked to the perceived presence of the supernatural [23].

Simultaneously, addressing culture-bound syndromes can influence and reduce mental health stigma across cultures. Misinterpretation of these syndromes can contribute to stigma, as individuals might be wrongly diagnosed or misunderstood. For instance, Kirmayer's (2012) study on cultural variations in depression and anxiety found that misunderstanding culture-bound syndromes, such as 'Taijin Kyofusho,' a Japanese syndrome characterized by an intense fear that one's body or bodily functions are displeasing to others, could lead to misdiagnosis and increase stigma [24]. Practices that raise awareness of culture-bound syndromes offer a deeper, richer perspective on cultural influences on mental health. Awareness and understanding of these syndromes can enhance diagnostic and treatment approaches, optimize patient outcomes, and potentially contribute to reducing mental health stigma across various cultures.

Taken together, these studies highlight the importance of understanding cultural contexts when addressing the stigma surrounding mental health disorders and psychiatric care. The cultural beliefs and attitudes towards mental health disorders, summarized below in Table 1, influence how stigma is manifested and the approaches needed to reduce it effectively. By acknowledging cultural variations, more culturally appropriate and effective strategies can be developed to combat stigma and improve mental health care across different societies worldwide.

**Table 1:** A Cultural Comparison on Mental Health Perception and Stigma

Authors	Cultural Group	Perception of Mental Illness	Impact on Stigma
Chen & Mak, 2008 [15]	Asian	Seen as a sign of personal weakness or failure of self-control	Stigma leads to family shame, discourages help-seeking
Girma et al., 2013 [17]	African	Attributed to spiritual or supernatural causes	High stigma levels, deter individuals from seeking psychiatric help
Karam et al., 2008 [20]	Arab	Viewed as a form of divine punishment	Significant stigma, leads to delayed or avoided treatment
Alegria et al., 2002 [21]	Latin American	Attributed to personal weakness or lack of willpower	Stigmatizes individuals, discourages them from seeking psychiatric care
Corrigan & Watson, 2007 [12]	Western	Misconceptions about danger or unpredictability	Results in social exclusion and discrimination

Strategies for addressing mental health stigma

Several strategies have been proposed in the literature to address the stigma surrounding psychiatry across cultures:

1. Public Awareness Campaigns

Awareness campaigns can be instrumental in dismantling misconceptions and fostering understanding of mental health disorders. Public awareness campaigns can dispel myths, reduce stigma, and encourage empathy towards affected individuals by promoting accurate information about mental illnesses, their prevalence, and the possibilities for recovery. For instance, a study by Pinfold et al., (2003) showed that public campaigns using direct social contact with people with mental illness could significantly improve public attitudes towards mental health [25]. The study by Pinfold et al., (2003) implemented educational interventions in UK secondary schools, consisting of video presentations and direct social contact with individuals who had personal experiences with mental illness [25]. The UK campaign's goal was to challenge common myths about mental illness and replace them with accurate information. The results showed that students exposed to this intervention demonstrated less fear and avoidance of people with mental health problems and were more likely to see them as individuals rather than defining them by their illness.

2. Cultural Competency Training for Healthcare Professionals

Medical education can equip healthcare providers with the necessary knowledge and skills to understand and respect their patients' cultural backgrounds and experiences, which is critical for reducing stigma in healthcare settings. Research indicates that healthcare providers who lack cultural competence may inadvertently contribute to stigma, further deterring patients from seeking help [26]. A study by Kirmayer (2012) found that cultural competence training improved healthcare providers' understanding of cultural influences on health behaviors and led to more effective patient-provider communication, thereby reducing perceived stigma [24]. For instance, a study in Australia provided cultural competency training to healthcare providers and found that their understanding of Indigenous Australians' health needs significantly improved [24]. They were able to better respect and incorporate Indigenous perspectives in treatment, which led to increased trust and better patient-provider relationships.

3. Peer Support Programs

People with lived experiences of mental health disorders who share their stories, can normalize mental health issues and challenge stigma. By providing real-life examples of individuals living with and managing their mental health disorders, peer-to-peer advocacy programs may debunk myths and reduce the perceived 'otherness' of mental illness. A study by Pitt et al. (2013) showed that peer support reduced self-stigma and improved self-esteem and empowerment among individuals with mental health disorders [27]. The study focused on "consumer-providers," individuals who had personally experienced mental health issues and were now providing support services to others. The findings demonstrated that consumer-providers significantly reduced self-stigma among service users, while also improving self-esteem and feelings of empowerment.

4. Community-Based Mental Health Services

Integrating mental health care into primary care and community settings can reduce the stigma associated with seeking psychiatric help. This emphasis on integrating measures for mental well-being along with other routine and standard primary care protocols allows mental health care to be more accessible and less intimidating, encouraging individuals to seek help when needed. A study by Thornicroft et al. (2015) found that community-based mental health services can reduce stigma and discrimination and improve mental health outcomes [28]. For instance, a program in India called the MANAS project integrated mental health services into primary care and community settings [28]. This approach not only made mental health services more accessible but also more 'normal' and less stigmatizing. The project reported a significant increase in the utilization of mental health services and a decrease in the experience of stigma among service users.

5. Evidence-Based Approach

Another approach to overcoming the barriers created by stigma is to use evidence-based methods to reduce mental illness stigma. A meta-analysis by Corrigan et al. (2016) found that various evidence-based interventions, including education and contact-based interventions, can effectively reduce mental illness stigma across cultures [9]. Contact-based interventions involve interaction between people with mental illness and members of the public to challenge negative attitudes and beliefs. Education-based interventions aim to increase knowledge and awareness of mental illness and reduce negative stereotypes. Educational interventions can be delivered in a variety of formats, such as in-person workshops, online courses, and mass media campaigns.

The role of the healthcare provider in ameliorating stigma cannot be overlooked. Moreover, a review by Ayalon and Areán (2004) suggests that mental health providers can play a critical role in reducing mental illness stigma by engaging in culturally sensitive practices [14]. For instance, mental health providers can develop cultural competence, which refers to the ability to provide effective services to individuals from diverse cultural backgrounds. Cultural competence involves understanding and respecting cultural differences, tailoring treatment to meet diverse populations' unique needs, and integrating cultural factors into treatment planning.

Research also highlights that stigma towards mental illness has significant implications for treating and managing mental health conditions. For example, several studies suggest that stigma can lead to delayed diagnosis and treatment-seeking behaviors [13,16]. This is concerning because early intervention is critical for managing mental illness and improving outcomes for individuals living with these conditions. Considering the documented impact of stigma on timely diagnosis and treatment-seeking behaviors, strategies such as public awareness campaigns, cultural competency training for healthcare professionals, peer support programs, community-based mental health services, and an evidence-based approach can play a crucial role in combating cultural stigma in psychiatry. These measures collectively contribute to improved awareness, understanding, and acceptance of mental health conditions, thus facilitating early intervention and better management of mental illnesses across diverse cultural contexts.

## Conclusions

Stigma surrounding mental health and psychiatric care is a complex and multifaceted issue that varies across ethnic and cultural contexts. To effectively address and reduce stigma in mental healthcare settings, developing culturally sensitive interventions and promoting understanding and acceptance of mental health issues is crucial. By doing so, we can work towards improving access to mental health care and promoting the well-being of individuals and communities across the globe.

Overall, the literature suggests that stigma is a complex and pervasive issue that affects individuals with mental illness across cultures. The studies reviewed reveal that mental illness stigma is influenced by cultural beliefs, attitudes, and values, and can manifest in different ways across cultures. It is important to understand these cultural differences to develop more effective interventions to reduce mental illness stigma and improve outcomes for individuals living with mental illness. Furthermore, stigma across cultures impacts psychiatric care in various ways and can create significant barriers to effective treatment. Evidence-based interventions, including education, contact-based interventions, and culturally sensitive practices can help overcome these barriers. Mental health providers should strive to develop cultural competence and deliver culturally sensitive interventions to meet the needs of diverse populations. Research to understand the impact of stigmatization of mental health patients and its impact in providing services is warranted. Reducing mental illness stigma is critical to providing equitable, effective, and compassionate psychiatric care to individuals with mental illness.
